# Unraveling the structure, chemical composition, and conserved signaling in leech teeth

**DOI:** 10.1080/19768354.2024.2350736

**Published:** 2024-05-11

**Authors:** Yam Prasad Aryal, Sanjiv Neupane, Hee-Jin Kwak, Chang-Hyeon An, Wern-Joo Sohn, Hitoshi Yamamoto, Tae-Yub Kwon, Bong-Ki Min, Jae-Young Kim, Sung-Jin Cho

**Affiliations:** a Department of Biological Sciences and Biotechnology, College of Natural Sciences, Chungbuk National University, Cheongju, Korea; b Department of Biochemistry and Cell Biology, Stony Brook University, Stony Brook, NY, USA; c Department of Oral and Maxillofacial Radiology, School of Dentistry, Kyungpook National University, Daegu, Korea; d Pre-Major of Cosmetics and Pharmaceutics, Daegu Haany University, Gyeongsan, Korea; e Department of Histology and Developmental Biology, Tokyo Dental College, Tokyo, Japan; f Department of Dental Biomaterials, School of Dentistry, Kyungpook National University, Daegu, Korea; g Center for Research Facilities, Yeungnam University, Gyeongsan, Korea; h Department of Biochemistry, School of Dentistry, Kyungpook National University, Daegu, Korea

**Keywords:** Leech, teeth, transcriptome analysis, conserved signaling, chemical nature

## Abstract

Unlike vertebrates, the number of toothed taxa in invertebrates is very few, with leeches being the only tooth-bearing organisms in the phylum Annelida. Copious studies have been conducted regarding vertebrate teeth; however, studies regarding the structure and function of invertebrate teeth are limited. In this study, the tooth structure of leeches, specifically *Hirudo nipponia* and *Haemadipsa rjukjuana*, was revealed, which showed sharp and pointed teeth along the apex of three jaws. Understanding conserved signaling regulations among analogous organs is crucial for uncovering the underlying mechanisms during organogenesis. Therefore, to shed light on the evolutionary perspective of odontogenesis to some extent, we conducted de novo transcriptome analyses using embryonic mouse tooth germs, *Hirudo* teeth, and *Helobdella* proboscises to identify conserved signaling molecules involved in tooth development. The selection criteria were particularly based on the presence of tooth-related genes in mice, *Hirudo* teeth, and *Helobdella* proboscis, wherein 4113 genes were commonly expressed in all three specimens. Furthermore, the chemical nature of leech teeth was also examined via TEM-EDS to compare the chemical composition with vertebrate teeth. The examination of tissue-specific genetic information and chemical nature between leeches and mice revealed chemical similarities between leech and mice teeth, as well as conserved signaling molecules involved in tooth formation, including *Ptpro*, *Prickle2*, and *Wnt16*. Based on our findings, we propose that leech teeth express signaling molecules conserved in mice and these conserved tooth-specific signaling for dental hard tissue formation in mice would corresponds to the structural formation of the toothed jaw in leeches.

## Introduction

Comparing intercellular signaling mechanisms across organisms is crucial for understanding developmental variations among species and elucidating the evolutionary processes driving morphological and functional changes (Reilly et al. 2015). Studies have shown that descendant animals share genomic features, developmental programs, and transcriptional regulatory cascades (Simakov and Kawashima 2017). Additionally, homologies in small subunit ribosomal RNA genes are utilized to establish evolutionary relationships in Deuterostomia and Protostomia (Bourlat et al. 2008). Consistent with this, the examination of the conserved genetic toolkit in animals through deep homology studies helps define fundamental processes for patterning novel structures (Schneider and Amemiya 2016). Although shared genomic content suggests developmental molecular similarities (Simakov and Kawashima 2017), the specific manifestation of these similarities in terms of morphological, structural, and functional aspects of organ development in distantly related organisms remains less explored.

Most vertebrates typically exhibit functional teeth characterized by hard tissues such as enamel, dentin, and cementum, with some exceptions observed in certain teleosts (Qu et al. 2015; Berkovitz and Shellis 2018). Similarly, certain invertebrates possess highly mineralized and functional teeth at the entrance of their alimentary canals, although such structures are not widespread among all invertebrates (Orevi et al. 2000; Koussoulakou et al. 2009). The medicinal leech *Hirudo*, a segmented protostome invertebrate belonging to the phylum Annelida, possesses a dorso-medial jaw and a symmetrical pair of ventro-lateral jaws, each featuring a single row of sharp teeth with a lateral aperture close to the tip (Orevi et al. 2000). Hence, it might be hypothesized that teeth were already present in the last common ancestor of vertebrates and leeches. However, phylogenetic evidence contradicts this interpretation. For instance, fossil records indicate that teeth emerged as an evolutionary novelty in jawless vertebrates (Koussoulakou et al. 2009). Among protostomes, toothed taxa are rare, even among leeches, as most species lack teeth. Therefore, it is assumed that teeth arose de novo within this group as well.

The evolution of tooth development is a subject of interest for both paleontologists and neontologists. In *Hirudo*, the primordial mouth develops in the anterior coalescent germinal plate, and this primordium eventually gives rise to oral structures such as the pharynx, oral epithelium, and jaws (Orevi et al. 2000). The teeth of leeches, embedded within the muscular jaw of the proboscis, differ from vertebrate teeth and are insufficient to illustrate the poorly understood dentition of non-chordates. This limitation severely restricts comparative studies of leech tooth-like structures with vertebrate teeth from an evolutionary perspective. However, conserved gene networks have been identified in the initiation of jaw and tooth development in vertebrates (Fraser et al. 2009). Therefore, to shed some light on the evolutionary perspective of odontogenesis, we conducted de novo transcriptome analysis of mouse, toothed leech (*Hirudo nipponia*) and toothless leech (*Helobdella austinensis*). We selected potential candidate genes for tooth formation based on commonly expressed genes in mice and leeches as revealed by transcriptome analyses, along with genetic information obtained from previous studies (Nishikawa and Kawamoto 2015; de Sousa-Romero and Moreno-Fernández 2016; Neupane et al. 2023; Song et al. 2023). Additionally, using electron microscopy, the present study revealed the chemical composition of leech teeth, along with identifying conserved signaling molecules associated with tooth formation.

## Materials and methods

### Animals

The experiments involving mice were performed in accordance with the guidelines of the Kyungpook National University, School of Dentistry, Intramural Animal Use and Care Committee (KNU-2020-0107). The time-mated pregnant C57BL/6 mice were purchased from Taconic, Korea and the embryos at stage 14 (E14) were used for tissue collection. Korean medicinal leeches *Hirudo nipponia,* were purchased from Leech farm (Medicinal Leech Distribution Company, Korea). *H. nipponia* (Order: Hirudiniformes, Family: Hirudinidae), also known as Korean blood-sucking leech, is an aquatic haematophagous leech which is widely distributed along the East-Asian countries including China, Japan, Korea, Mongolia, and eastern Russia (Wang et al. 2022). *Haemadipsa rjukjuana* were collected from the Gageo island, Jeollanam-do, South Korea. *H. rjukjuana* (Order: Hirudiniformes, Family: Haemadipsidae) is a terrestrial haematophagous leech found in east and southeast-Asian countries including Japan, China, Malaysia, Indonesia, Taiwan and South Korea (Lai et al. 2011; Won et al. 2014). The maintenance, living conditions, and experimental protocols of *Hirudo* and *Haemadipsa* were performed at the Chungbuk National University. *Helobdella austinensis* were artificially bred in an incubator at 22 °C. The culture and embryo collections of *Helobdella* were performed as previously described (Weisblat and Kuo 2009).

### Harvesting of mouse and leech tissues

Embryonic (E14) mandibles of C57BL/6 mice were dissected in RNase-free conditions and processed for laser microdissection (LMD) (Leica Microsystems, Germany; LMD 6000) as previously described (Neupane et al. 2023). Since the primary enamel knot (PEK) is considered an important signaling center for tooth development (Thesleff and Jernvall 1997; Thesleff et al. 2001), only the PEK tissue was collected via LMD, and then RNA was extracted for transcriptome analysis. Similarly, *Hirudo* teeth were dissected using surgical scissors, and the proboscises of *Helobdella* were dissected using sharp insect pins. RNA was extracted from *Hirudo* teeth and *Helobdella* proboscises using TRIzol (Ambion, Austin, USA), as previously described (Kwak et al. 2022).

### Scanning electron microscopy (SEM) and transmission electron microscopy (TEM)

SEM and TEM of leech jaw apparatus was performed according to standard operating protocols, as described previously (Khan et al. 2014). The leech jaw specimens were scanned for teeth-like surface structure and morphology using SEM (JSM-6700F, Jeol, Tokyo, Japan). Additionally, they were investigated by TEM (Titan G2 ChemiSTEM, FEI, USA) equipped with an Energy-dispersive X-ray spectroscopy (EDS), for microstructure and chemical composition. Nano-thin lamellae for TEM were prepared by focused ion beam (FIB; Versa3D LoVac, FEI, USA) sectioning using Ga3+ ions with milling and polishing. After final polishing, the lamellae were lifted-off using a tungsten tip (Omni Probe 400; Oxford Instruments, UK) and attached to a sample grid for TEM analysis.

### RNA-seq library preparation, sequencing, and preprocessing

The total RNA concentration and its integrity were evaluated by Agilent 2100 Bioanalyzer (Agilent, CA, USA). A library was independently prepared using 1μg of total RNA for each sample using the Illumina TruSeq RNA Sample Prep Kit v2 (Illumina, Inc., San Diego, CA, USA) following the manufacturer’s instructions (Part# 15026495 Rev. F). The paired end (2 × 101 bp, 2 × 151 bp) sequencing was performed by Macrogen Incorporated on the Illumina HiSeq 4000 platform (Illumina, Inc., San Diego, CA, USA). After the sequencing run, the raw reads from specimens were trimmed by preprocessing using Trimmomatic v0.33 (Bolger et al. 2014) using the default parameters except for MINLEN, which is the minimum read length with 36 and 50 bp for 101 and 151 bp paired end reads, respectively.

### Transcriptome analysis

For mouse, clean reads were aligned to the mouse reference genome by Tophat v2.1.0 under the default parameter settings (Kim et al. 2013). The mapped read and annotated transcriptome information were fed to Cufflinks v.2.2.1 for transcript assembly and expression level quantification (Trapnell et al. 2010). Next, the calculated FPKM (Fragments Per Kilobase of transcript per Million mapped reads) per genes were converted to TPM (Transcript per Million) using the formula: (FPKM of genes / ∑FPKM) × 106 ( Li and Dewey 2011). The mouse genome and annotation data (mm10) were obtained from the UCSC genome browser (https://genome.ucsc.edu). Because of the lack of a reference genome, preprocessed reads of *Hirudo* teeth and *Helobdella* proboscis were independently de novo assembled using Trinity v.2.1.1 with default parameter settings (Grabherr et al. 2011). After assembly, functional protein coding sequences (CDS) were predicted by TransDecoder v.3.0.0 (http://transdecoder.sourceforge.net). To maximize the ability of capturing CDS, all assembled transcripts were performed by homology-search interrogating the Uniprot/Swiss-Prot database (http://www.uniprot.org) via BLASTP with an E-value threshold 10-5. Short CDSs with lengths shorter than 100 amino acids were discarded. Highly identical transcripts were clustered and removed without longest one using CD-HIT v.4.6.5 (Fu et al. 2012) with default parameters except for -c 0.99. Next, we generated non-redundant CDS (NRCDS) for each specimen. To annotate the NRCDS, these were compared and input into the Uniprot/Swiss-Prot database though BLASTP with an E-value cutoff 10-10 and the best blast hit. For multiple CDS that mapped to different proteins in the database, the longest CDS was assigned first to that protein. To quantify gene expression levels of *Hirudo* teeth and *Helobdella* proboscis, clean reads were aligned separately to the reference transcriptome and the estimated TPM was determined using Bowtie v.2.2.6 (Langmead and Salzberg 2012) and RSEM 1.2.26 (Li and Dewey 2011), respectively.

### Gene alignment and phylogenetic analysis (*Prickle2, Ptpro,* and *Wnt16*)

Using Blastx and ExPASy (Swiss Institute of Bioinformatics), we filtered the shifted frames and obtained the correct amino acid sequences that were translated from the analyzed transcriptomes. The amino acid orthologs of *Prickle2, Ptpro*, and *Wnt16* from representative species sample (mouse teeth, *Hirudo* teeth, *Helobdella* proboscis) sequences were aligned using the ClustalW2 program, and were visualized using a Bioedit (http://www.mbio.ncsu.edu/BioEdit/bioedit.html).

### Histomorphology

*Hirudo nipponia* and *Haemadipsa rjukjuana* were dissected in phosphate buffer saline (PBS) to expose the jaws under translucent microscope (Leica S9E). The histomorphological structures of adult leech and jaws were imaged using LEICA DM6 B microscope. Similarly, *Helobdella austinensis* were dissected in PBS to expose the proboscis. For *Hirudo* and *Haemadipsa*, the single jaws were separated and fixed in 4% paraformaldehyde (PFA), washed with PBS, treated with 15% and 30% sucrose solution, and embedded in optimal cutting temperature compound (OCT) (Surgipath). Then after, cryocut of 10 µm thickness were prepared for histological analysis. Histomorphological analyses were performed using hematoxylin and eosin (H&E) staining as described previously (Neupane et al. 2023).

### In situ hybridization (ISH)

To examine the expressions of odontogenic genes, ISH was performed as described previously (Cho et al. 2010; Kwak et al. 2022). The details of digoxigenin-labeled RNA probes are provided in Table S1. The hybridization temperature was maintained at 64.7°C. A 1% Anti-DIG solution in 1X PBS was used as blocking reagent. The color reaction was performed using nitro blue tetrazolium chloride/ 5-bromo-4-chloro-3-indoyl-phosphate (Roche, Basel, Switzerland) by standard procedures. Stained samples were imaged using a LEICA DM6 B with a LEICA DFC450 C camera (Leica, Wetzlar, HE, Germany). The obtained images were edited using Las X software (Leica, Wetzlar, HE, Germany).

## Results

### Morphology of leech teeth

**Figure 1.**
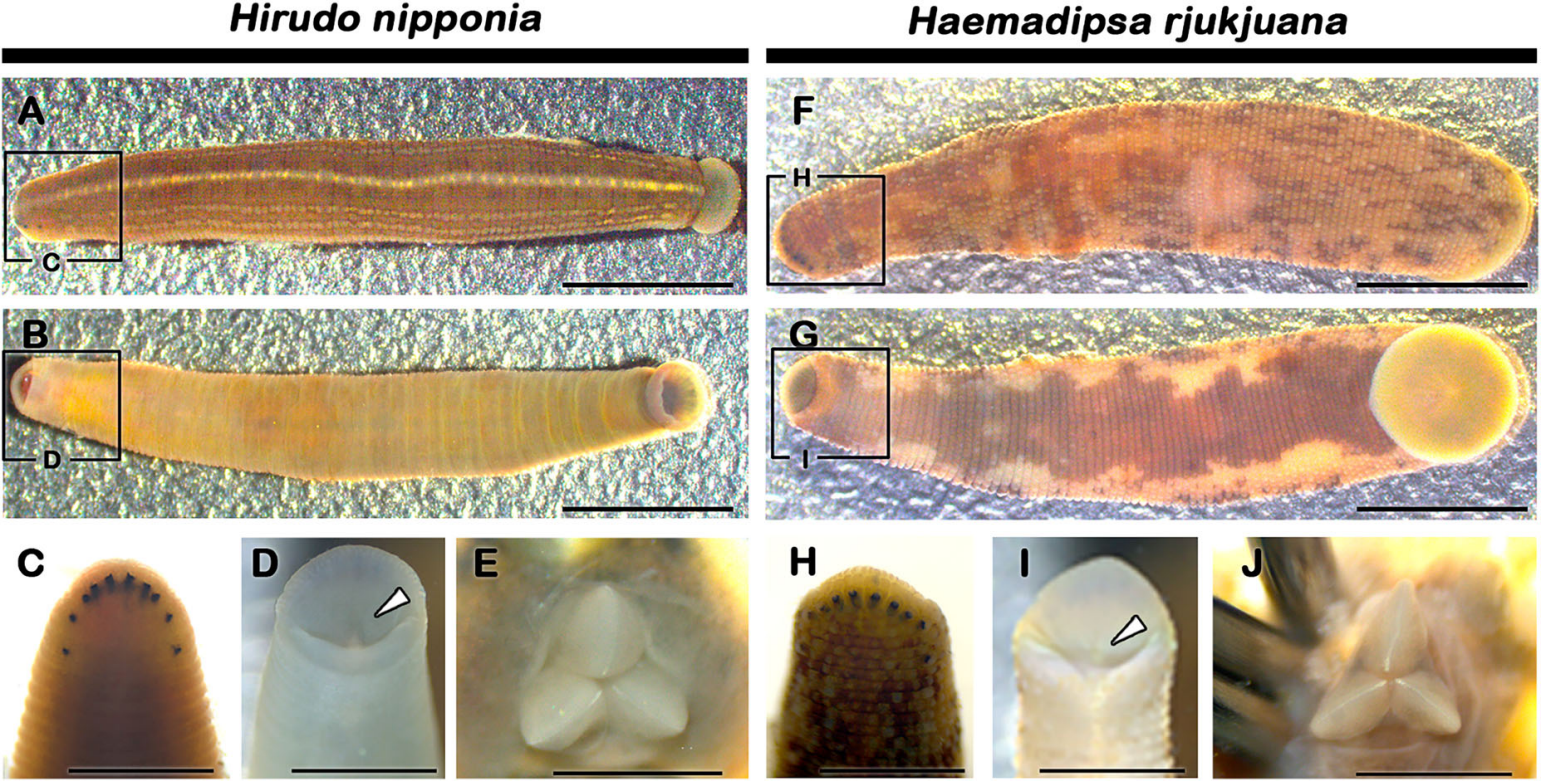
Morphology of adult leeches. Dorsal (A) and ventral (B) view of adult *Hirudo*. Magnified dorsal view of *Hirudo* showing five pairs of eyes (C). Fourth and fifth eyespot pairs are separated by two annuli (C). Anterior sucker of *Hirudo* showing location of jaws (white arrowhead) (D). Magnified view of jaws of *Hirudo* (E). Dorsal (F) and ventral (G) view of adult *Haemadipsa*. Magnified dorsal view of *Haemadipsa* showing five pairs of eyes (H). Fourth and fifth eyespot pairs are separated by two annuli (H). Anterior sucker of *Haemadipsa* showing location of jaws (white arrowhead) (D). Magnified view of jaws of *Haemadipsa* (J). Boxes in A, B, F and G are magnified views in C, D, H and I respectively. Scale bars: 5 mm (A–B, F–G), 3 mm (C–D, H–I), 1 mm (E, J).

The bodies of *Hirudo nipponia* and *Haemadipsa rjukjuana* are elongated and cylindrical, each consisting of anterior and posterior suckers (Figure 1(A–J)). The dorsal body surface of *Hirudo* is blackish-green with five continuous yellowish longitudinal stripes extending from the anterior to the posterior end of the body (Figure 1(A)). Among these stripes, the median one appears comparatively thicker than the other lateral stripes (Figure 1(A)). Conversely, the dorsal body surface of *Haemadipsa* is yellowish or grayish-brown, adorned with irregularly distributed black spots (Figure 1(F)). Unlike *Hirudo*, *Haemadipsa* lacks a median stripe on the dorsal part of the body (Figure 1(F)). In both species, the anterior part of the body comprises five pairs of eyes arranged bilaterally in the second, third, fourth, fifth, and eighth annulus (Figure 1(C, H)). The fourth and fifth pairs of eyespots are separated by two annuli, while the other four pairs are continuous from the second to the fifth annuli (Figure 1(C, H)). The anterior sucker, characteristic in both species, is wide, triangular, and cup-shaped, housing three jaws as a mouth apparatus (Figure 1(D, E, I, J)). Among the three jaws, one is positioned antero-dorsally, while the other two are situated lateroventrally (Figure 1(E, J)). Each jaw possesses a single row of denticles at the apex (monostichodont) (Figure 1(E, J)). Two rows of teeth converge at the denticle tips, forming a single row in both species (Figure 1(E, J)). In *Haemadipsa*, nearly half of the jaw apex features teeth, while in *Hirudo*, teeth consistently appear from the mesial to the distal part of the jaw apex (Figure 1(E, J)). Furthermore, the jaws of *Haemadipsa* exhibit more densely packed teeth compared to *Hirudo* (Figure 1(E, J)). Statistically, the number of teeth in each jaw of *Haemadipsa* was comparatively fewer than that of *Hirudo*. The average number of teeth per jaw in *Hirudo* was found to be 65, whereas in *Haemadipsa*, the average was 46 (Figure 1(E, J)).

### Histology of leech jaw and teeth

**Figure 2.**
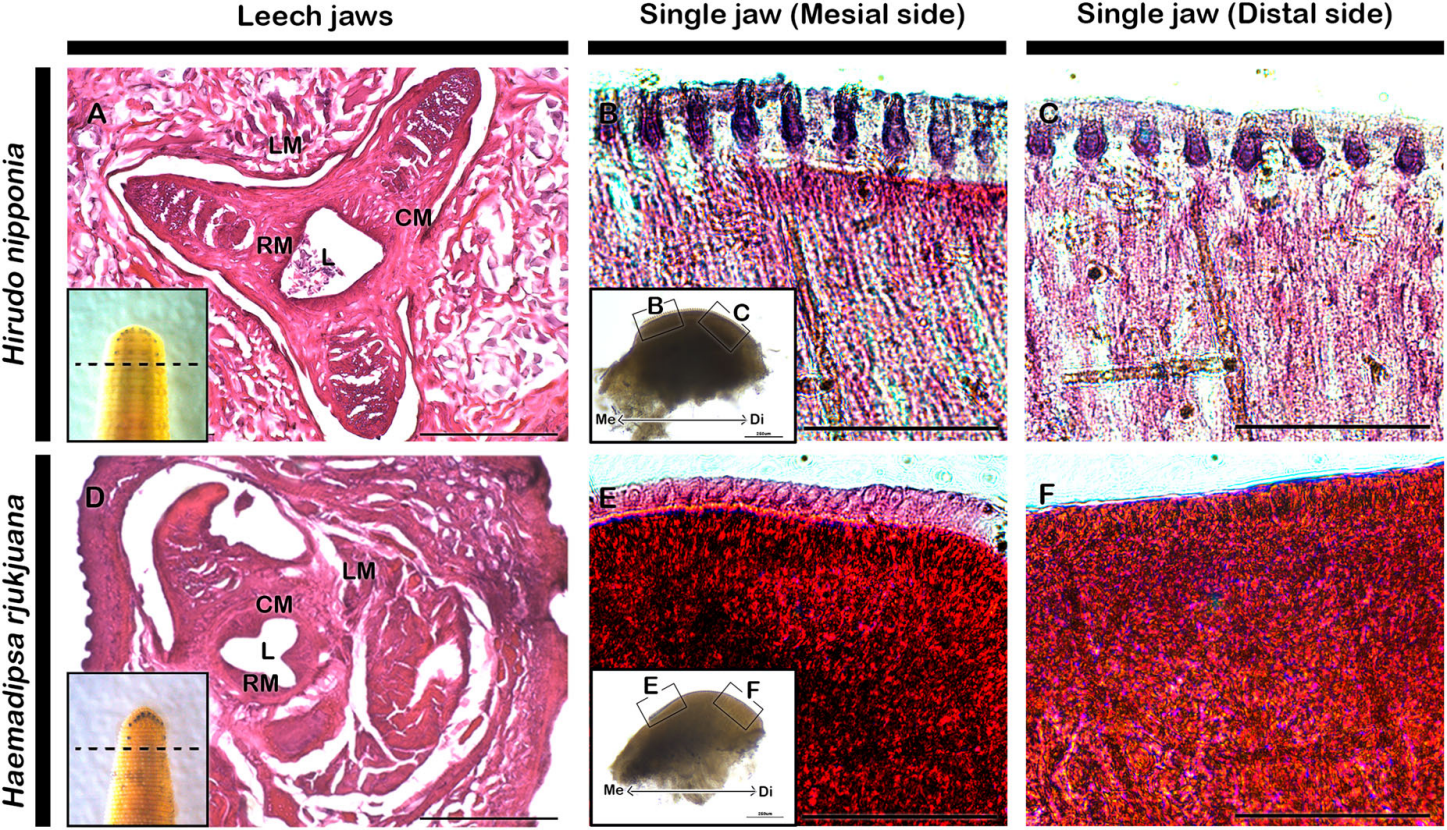
Histology of leech jaw. H&E staining showing cross sections of the leech jaw displaying three jaws surrounded by radial muscle, circular muscle and longitudinal muscle in both species (A, D). H&E staining of the longitudinal sections of the single jaw of adult leech (B–C, E–F). L.S. of jaw of *Hirudo* showing sharp, pointed and pyramidal-shaped tooth on the apex of mesial and distal part of jaw (B–C). L.S. of jaw of *Haemadipsa* showing pointed and conical-shaped teeth on the apex of mesial side of jaw, while absence of teeth in the distal side of jaw (E–F). Insets in A and D indicate cross section levels. Insets in B and E are section levels. L, lumen; CM, circular muscle; RM, radial muscle; LM, longitudinal muscle; Me, mesial; Di, distal. Scale bars: 500 µm (A, D), 100 µm (B–C, E–F).

Both leeches are characterized by three jaws located at the anterior part of the body. Each jaw appears as a crescent-shaped structure and is connected together with a lumen (Figure 2). In *Hirudo*, the lumen has a roughly triangular shape, whereas in *Haemadipsa*, it curves inward, forming a roughly Y-shaped structure (Figure 2(A,D)). The transverse section of the leech jaw revealed the presence of circular, radial, and longitudinal muscles surrounding the lumen in the anterior part of the body (Figure 2(A,D)). To examine the tooth structure in detail, we conducted longitudinal sections of the individual jaw, observing the tooth structure and its adjacent tissue from the mesial to the distal part of the jaw. In *Hirudo*, the teeth appeared sharp, pointed, and pyramidal in shape, firmly attached to the jaw muscle (Figure 2(B, C)). The part of the jaw facing toward the lumen is termed as mesial, while the part of the jaw facing toward the lateral side is termed as the distal side (Insets B, E). Teeth on the mesial side are comparatively longer and slender compared to those on the distal part (Figure 2(B, C)). In contrast, the teeth in *Haemadipsa* are sharp and roughly conical-shaped, also attached to the jaw muscle (Figure 2(E, F)). The teeth are densely packed with little gap between each tooth compared to *Hirudo* (Figure 2(E, F)). Interestingly, the distal part of the jaw in *Haemadipsa* lacks teeth, featuring only a smooth muscular layer (Figure 2(F)). When comparing the structure and arrangement of teeth between two leeches, the teeth of *Hirudo* appear more distinct and uniformly arranged on the jaw apex. Therefore, the functional study was performed using *Hirudo* teeth as a representative sample in the present study.

### Chemical nature of *Hirudo* teeth

**Figure 3.**
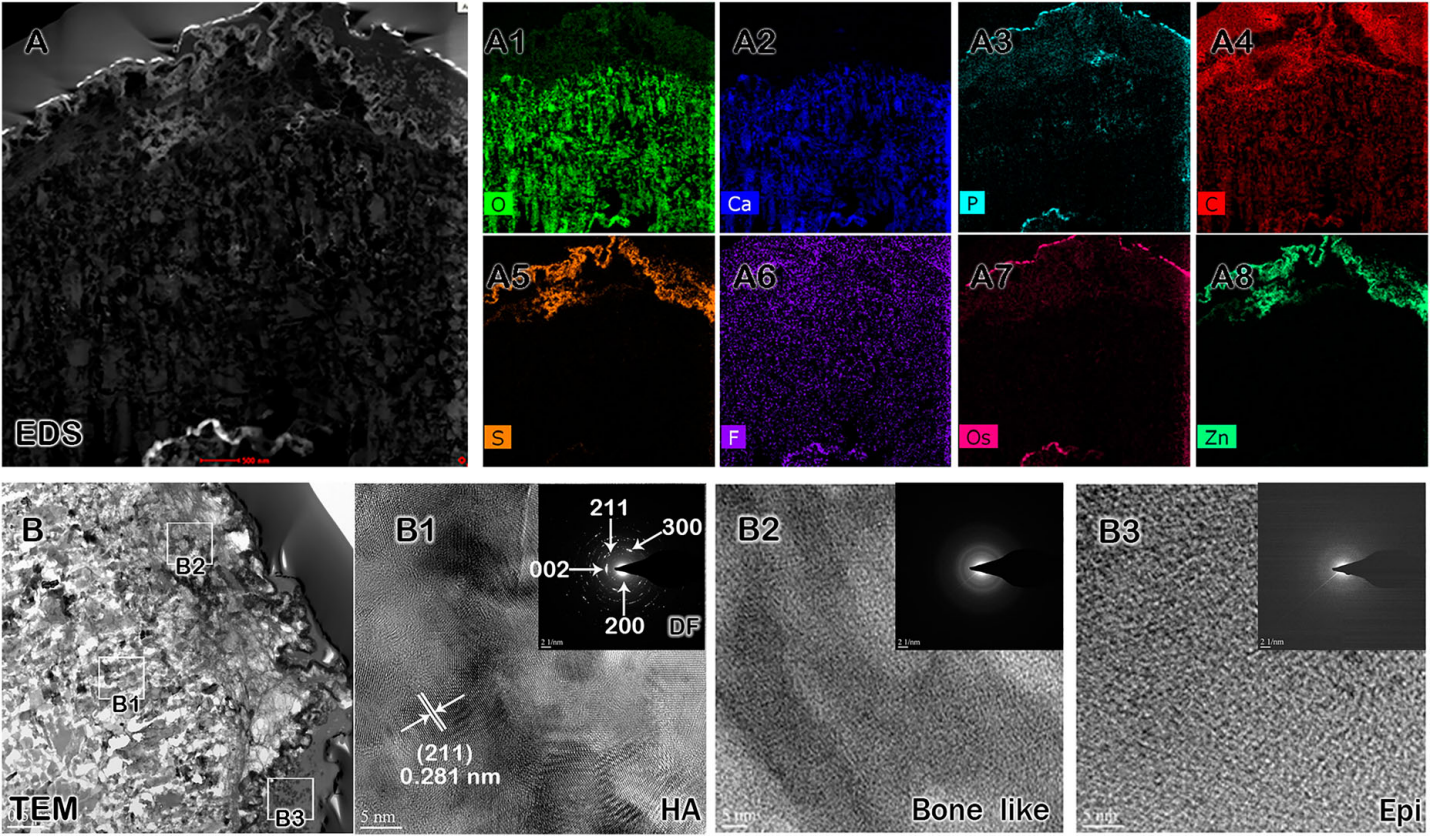
Chemical composition of *Hirudo* teeth. Elemental analysis of *Hirudo* teeth using EDS and TEM (A–B). Elemental analysis by EDS shows the presence of Calcium, Oxygen, Carbon, Phosphorus, Zinc, Osmium, Fluoride, and Sulfur (A, A1–A8). TEM analysis of different regions of the *Hirudo* tooth (B) shows hydroxyapatite structures (B1), bone-like structures (B2), and epidermal surface (B3). HA, hydroxyapatite; Epi, epidermal tissue. White boxes indicate the regions in the *Hirudo* tooth for ultrastructural analysis (B). Scale bars: 0.5 µm (A), 5 nm (B1–B3).

The presence of sharp rows of uniformly arranged and pointed teeth in *Hirudo*, the structure and arrangement of which can be comparable to those of some vertebrates (Bemis et al. 2005; Jambura et al. 2018), prompted us to examine the chemical nature of leech teeth to determine whether they show similarities to vertebrate teeth. For this purpose, we conducted TEM-EDS to map the spatial distribution of elements (Figure 3(A)). Our results showed the presence of Carbon, Oxygen, Calcium, Phosphorus, Sulfur, Osmium, Fluoride, and Zinc in *Hirudo* teeth (Figure 3(A1–A8)). Moreover, TEM analysis compared the crystallinity of bone-like hydroxyapatite and epidermal tissue (Figure 3(B)). The tooth surface exhibited both crystalline (Figure 3(B1)) and amorphous structures (Figure 3(B2, B3)). Furthermore, the selected area diffraction pattern (SAED) confirmed the presence of apatite and a highly polycrystalline structure along the tooth surface of *Hirudo* (Figure 3(B1–B3)).

### Transcriptome analysis and expression of tooth-related signaling molecules

**Figure 4.**
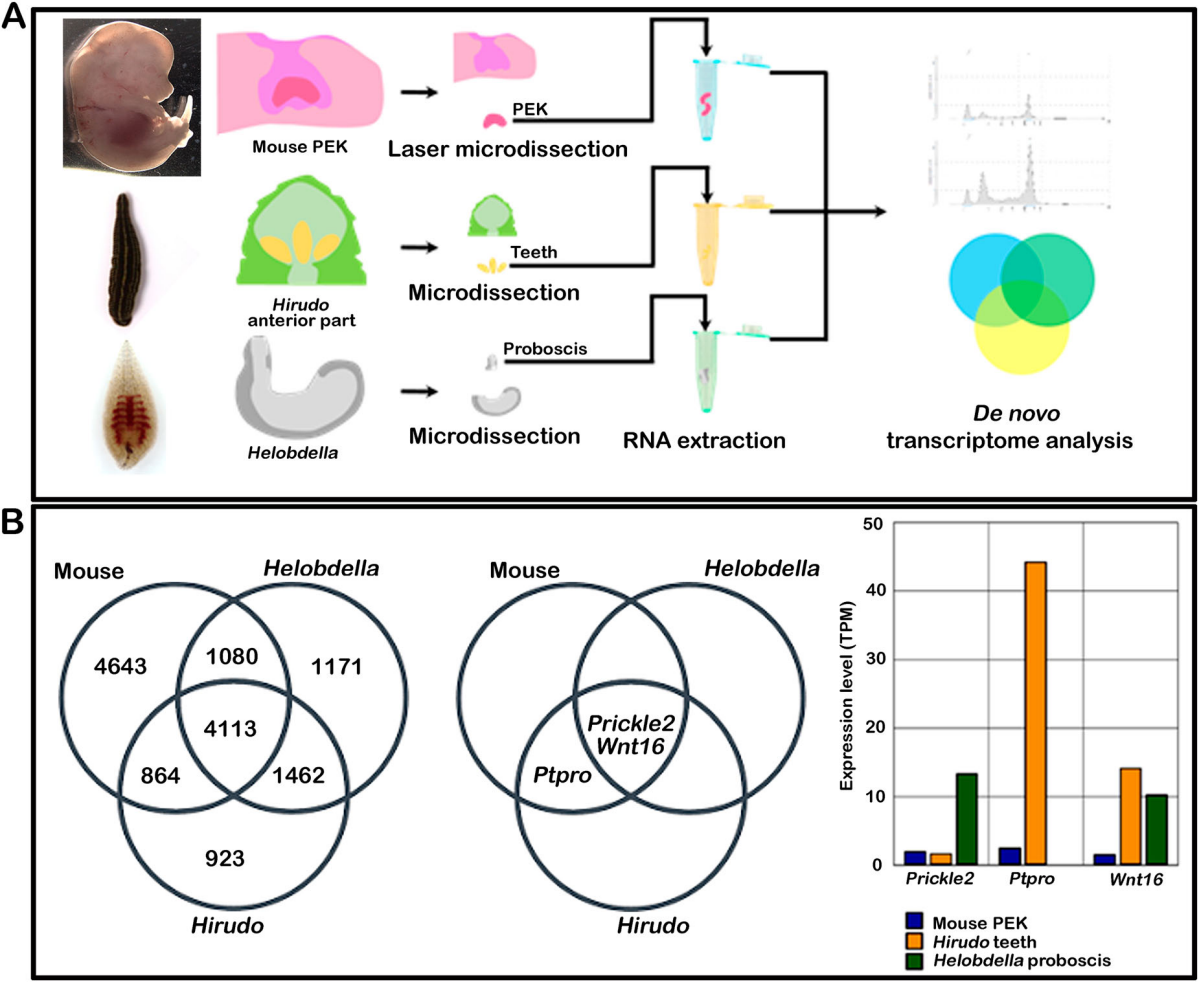
Transcriptome analysis for identification of tooth-related signaling molecules. The scheme of tissue collection from mice mandibles, *Hirudo* teeth, and *Helobdella* proboscises subjected to de novo transcriptome analysis (A). Venn diagrams showing the number of genes common in mouse teeth, *Hirudo* teeth, and *Helobdella* proboscises after extensive analysis (B). *Prickle2* and *Wnt16* are common to all species; *Ptpro* is common to mouse and *Hirudo* teeth (B). Bar diagram showing the expression levels (in TPM) of three genes: *Prickle2, Ptpro*, and *Wnt16* (B).

**Figure 5.**
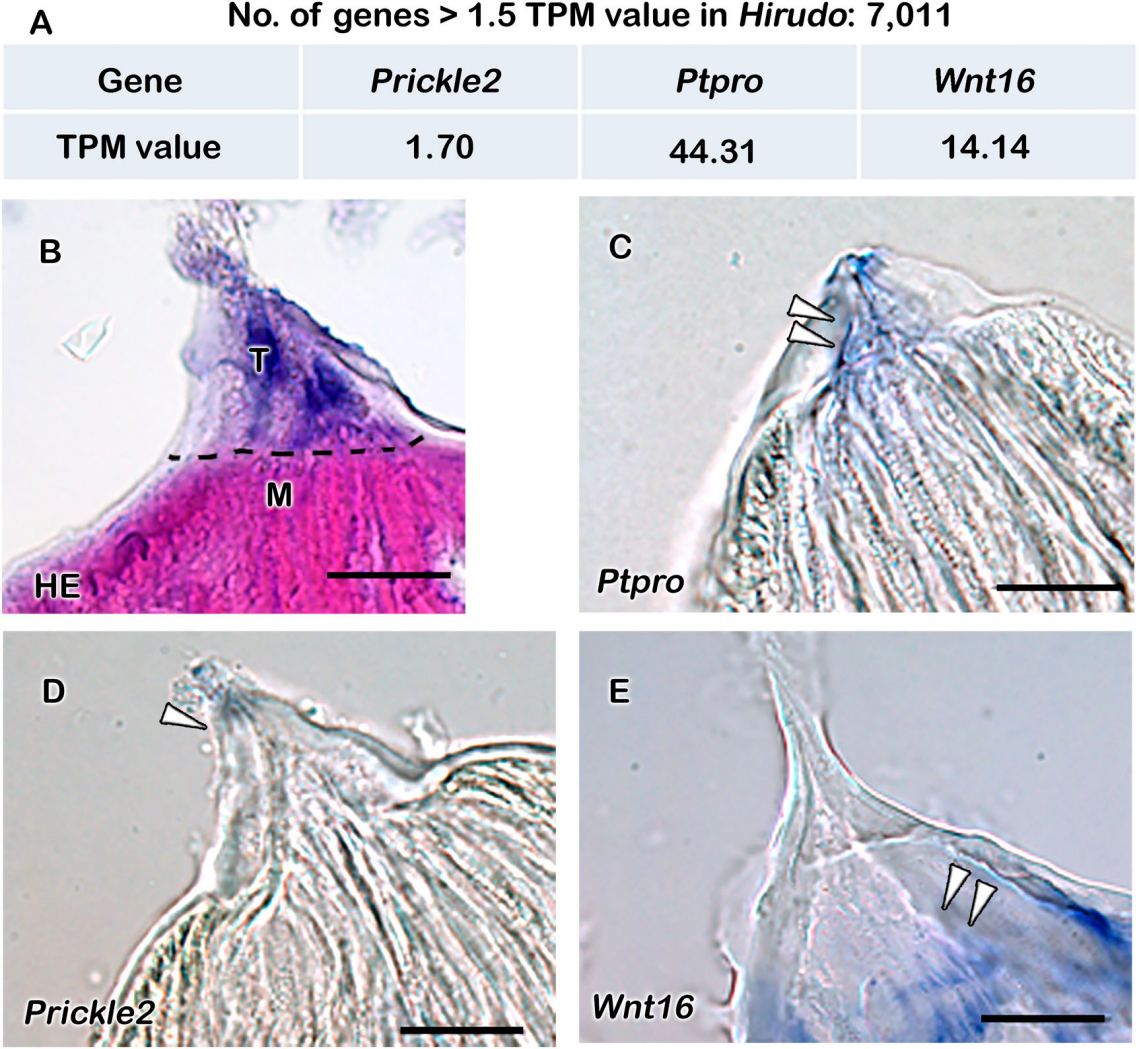
Expressions of tooth-related signaling molecules. Table showing number of genes with >1.5 TPM value in *Hirudo* teeth after extensive transcriptome analysis (A) and TPM value of the selected genes (A). Transverse section of *Hirudo* jaw showing pointed teeth embedded in the jaw muscles by H&E staining (B). In situ hybridization showing expressions of tooth-related signaling molecules: *Ptpro*, *Prickle2* and *Wnt16* in the *Hirudo* teeth and its surrounding tissue (C–E). The transverse section of a single jaw showing specific expression pattern of *Ptpro* from base to the tip of teeth (C). *Prickle2* is weakly expressed along the teeth and adjacent muscle, whereas intense expression of *Wnt16* is observed at the base of the teeth and adjacent muscle (D–E). Blue color indicates gene expression patterns (Arrowheads, C–E). Dotted line in B demarcates teeth and muscle boundary. T, teeth; M, muscle; HE, hematoxylin and eosin. Scale bar: 20 µm (B–E).

After examining the histomorphology and chemical nature of leech teeth, it led us to examine whether leech teeth possess conserved signaling molecules involved in vertebrate tooth formation. For this purpose, we selected three organisms: the toothed leech (*Hirudo nipponia*), the toothless leech (*Helobdella austinensis*), and the toothed vertebrate (Mouse, *Mus musculus*). Subsequently, RNA transcriptome analyses were performed using mouse tooth germs, *Hirudo* teeth, and *Helobdella* proboscises (Figure 4(A)). The transcriptome data from the toothless leech, *Helobdella*, were analyzed to deduce the signaling molecules present in toothless leeches. Given the absence of high-quality genome assembly for non-model organism, we adopted our previously published de novo transcriptome analysis pipeline (Park et al. 2018) for *Hirudo* teeth and *Helobdella* proboscis. As a result of the analysis, we obtained de novo transcriptome assembly consisting of 145,066 and 109,172 transcripts with assembly N50 values as 1639 and 1381bps, for tooth bearing and toothless leech respectively. To deduce the functional elements, protein coding regions were predicted for all transcripts and redundant transcripts were discarded. We identified 29,726 and 31,674 non-redundant protein coding sequences (NRCDS) and estimated their expression levels as transcript per million (TPM). Among these, 8003 and, 7968 NRCDS were assigned to the Uniprot/SwissProt database which is set of manually annotated proteins for *Hirudo* teeth and the *Helobdella* proboscis, respectively (Figure 4(B), Dataset 1). For the mouse teeth, RNA-seq data were separately analyzed using the Tuxedo suit including TopHat and Cufflinks tools (Trapnell et al. 2012). All mouse gene symbols were assigned to Uniprot/SwissProt id, to directly compare transcriptomes of the mouse and two non-model organisms. When we compared transcriptomes of mouse teeth, *Hirudo* teeth, and *Helobdella* proboscis, we identified 4,113 genes commonly expressed by all three samples (Figure 4(B), Dataset 1). Specifically, genes shared with the mouse showed 67.6% (4113 + 864/7362) and 66.3% (4113 + 1080/7826) in *Hirudo* teeth and *Helobdella* proboscis, respectively (Figure 2(B), Dataset 1). From these datasets, we selected potential candidate genes for tooth formation based on specific criteria: (i) expression in tooth-forming tissues in mice and *Hirudo* teeth (Figure 4(B); Nishikawa and Kawamoto 2015; de Sousa-Romero and Moreno-Fernández 2016; Neupane et al. 2023; Song et al. 2023) and (ii) lack of expression in the toothless leech (*Helobdella* proboscis). Following these criteria, we identified three genes – protein tyrosine phosphatase receptor type O (*Ptpro*), prickle planar cell polarity protein 2 (*Prickle2*), and wnt family protein 16 (*Wnt16*) (TPM > 1.5) (Figure 3(A) and Figure 4(B)). *Ptpro* was expressed in both mice and *Hirudo* teeth but not in *Helobdella* proboscis, whereas *Prickle2* and *Wnt16* were expressed in all species (Figure 4(B) and Figure 5(A)). Moreover, the protein alignment of PTPRO, PRICKLE2, and WNT16 revealed a highly conserved phosphatase domain across various animals, including arthropods, annelids, fish, amphibians, birds, and mammals (Figure S1). These results suggest that *Ptpro, Prickle2*, and *Wnt16* are conserved genes that likely play roles in dental hard tissue formation across metazoans, including leeches.

Furthermore, to examine the expression patterns of *Ptpro, Prickle2*, and *Wnt16* in *Hirudo* teeth, we conducted in situ hybridization (Figure 5(B–E)). The transverse section of a single jaw displayed specific *Ptpro* expression from the base to the tip of the teeth (Figure 5(B,C)). Conversely, the expression pattern of *Prickle2* appeared weaker along the teeth and adjacent muscle, while intense expression of *Wnt16* was observed at the base of the teeth and adjacent muscle (Figure 5(B, D–E)).

## Discussion

Teeth are the hardest calcified structures present in animals (Tummers and Thesleff 2009) and are thought to have evolved from pharyngeal denticles or dermal armors (McCollum and Sharpe 2001). Among annelids, leeches are the only tooth-bearing organisms. Since most invertebrate organisms lack teeth, it is still difficult to understand the development of dental hard tissue formation from an evolutionary perspective. In this study, we examined the tooth structures of two leeches: *Hirudo nipponia* and *Haemadipsa rjukjuana*. Our results showed sharp, pointed, and backwardly directed teeth in both species (Figure 1), however, the *Hirudo* teeth exhibited more distinct morphological features than other species, prompting us to conduct a detailed functional study. The continuous presence of sharp and pointed teeth along the apex of the jaw in *Hirudo* is likely an adaptive feature that enables effective and secure piercing of the skin of aquatic hosts while maintaining grip, due to the increased resistance in aquatic environments compared to terrestrial settings. Using *Hirudo* as a model organism, we employed an evolutionary developmental biology approach to understand whether the teeth in non-chordates exhibit similarities with vertebrate teeth. We used embryonic mice tooth germs, *Hirudo* jaws, and *Helobdella* proboscises to examine conserved signaling pathways for tooth development for the first time, employing de novo transcriptome analysis. This aimed to better understand the development of dental tissue in non-chordate animals and compare it with mice teeth (Figure 4; Nishikawa and Kawamoto 2015; de Sousa-Romero and Moreno-Fernández 2016; Neupane et al. 2023; Song et al. 2023). The transcriptome data from the toothless leech, *Helobdella*, was analyzed to deduce the signaling molecules present in toothless leeches. The extensive analysis of leech teeth revealed that they shared similar morphology and chemical composition with mice teeth (Figure 1–3), consistent with previous findings (Lewis and Nyman 2008; Šepitka et al. 2012).

The high-throughput analysis of leech teeth and proboscis revealed significant numbers of odontogenic genes shared with vertebrate tooth development (Figure 4; Nishikawa and Kawamoto 2015; de Sousa-Romero and Moreno-Fernández 2016; Neupane et al. 2023; Song et al. 2023). A previous study demonstrated deep genetic homology in specific tissue development among bilaterians (Tarazona et al. 2016), suggesting possible homology between mouse and *Hirudo* teeth regarding genetic factors. Our RNA transcript data unveiled the presence of *Prickle2* and *Wnt16* genes common to all species, while *Ptpro* was exclusive to mice and *Hirudo* teeth (Figure 2). This suggests that *Ptpro* was recruited in *Hirudo* but not in *Helobdella* spp., likely playing a crucial role in tooth development compared to *Prickle2* and *Wnt16*, as indicated in a previous report (Neupane et al. 2023). Furthermore, our elemental composition analysis confirmed that *Hirudo* teeth not only possess a structurally analogous composition to vertebrate teeth but also contain a chemical makeup similar to terrestrial organisms (Zenóbio et al. 2011), potentially resembling primitive forms of enamel. Calcium phosphate is the major mineral component of terrestrial vertebrate hard tissues and particularly, the teeth are made from the molecules of hydroxyapatite (HA), fluoroapatite (FA) and carbonate hydroxyapatite (de Dios et al. 2015; Dehghani Nazhvani et al. 2019). A previous study hypothesized the likely presence of hydroxyapatite in *Hirudo* teeth (Šepitka et al. 2012), and our EDS result indeed revealed the presence of hydroxyapatite crystals (Figure 3).

The knockdown of *Ptpro* resulted in retarded tooth development, while overexpression facilitated the process (Neupane et al. 2023), and *Prickle2* and *Wnt16* have been reported to be involved in dental hard tissue development and morphogenesis (Nishikawa and Kawamoto 2015; de Sousa-Romero and Moreno-Fernández 2016; Song et al. 2023). Additionally, *Prickle2* has been implicated in neurite formation during brain development (Okuda et al. 2007), enamel rod decussation (Nishikawa and Kawamoto 2015), the Wnt and planar cell polarity pathway, and pluripotency (Tao et al. 2012), suggesting that *Prickle2* and *Wnt16* might share similar signaling functions during tooth and proboscis formation in leeches. Meanwhile, the developmental roles of *Wnt16* in bone formation, human epidermal keratinocyte proliferation and differentiation (Teh et al. 2007), dental pulp stem cell differentiation (Takeda et al. 2008), and hematopoietic stem cell specification (Clements et al. 2011) have been well described, indicating that *Wnt16* could be involved in the differentiation of various tissues, including cells forming hard tissue. Our previous studies have also shown that *Wnt16* undergoes duplication in the lineage leading to leeches (Cho et al. 2010). It can be inferred that irrespective of toothed jaw or proboscis, conserved signaling molecules for hard tissue formation would play a role in creating tooth-like hard tissue in *Hirudo* and needle-like protrusible proboscis in *Helobdella*. *Hirudo* uses its toothed jaws to feed on blood from the host. In toothless leeches, the proboscis serves as the ingesting organ during attachment to the host for a blood meal, which could be analogous to the toothed jaw of *Hirudo* in terms of feeding and attachment (Weisblat and Kuo 2009; Kwak et al. 2022). Therefore, the expression of dental hard tissue-specific genes in the proboscis of toothless leeches would suggest functional similarity.

Our findings indicate that the signaling regulations involved in mammalian tooth development are conserved beyond mammals. *Hirudo* teeth express signaling molecules that are conserved in mice. These conserved signaling molecules, including *Ptpro, Prickle2*, and *Wnt16*, are likely involved in dental hard tissue formation by potentially modulating signaling pathways during odontogenesis. To gain a better understanding of dental hard tissue formation from an evo-devo approach, it is essential to study dental hard tissues in other non-chordate animal models in the near future.

## Supplementary Material

Supplemental Material

## Data Availability

All data generated for the present study are included in the manuscript. The datasets used during the current study are available from the corresponding author upon reasonable request.
