# Supplementary material for: Unraveling the structure, chemical composition, and conserved signaling in leech teeth
Source: Anim Cells Syst (Seoul). 2024 May 11;28(1):272–82. doi: 10.1080/19768354.2024.2350736 (PMC11089927; doi:10.1080/19768354.2024.2350736)
Supplement: Supplemental Material [file TACS_A_2350736_SM1770.docx]

**Supplementary files**

**
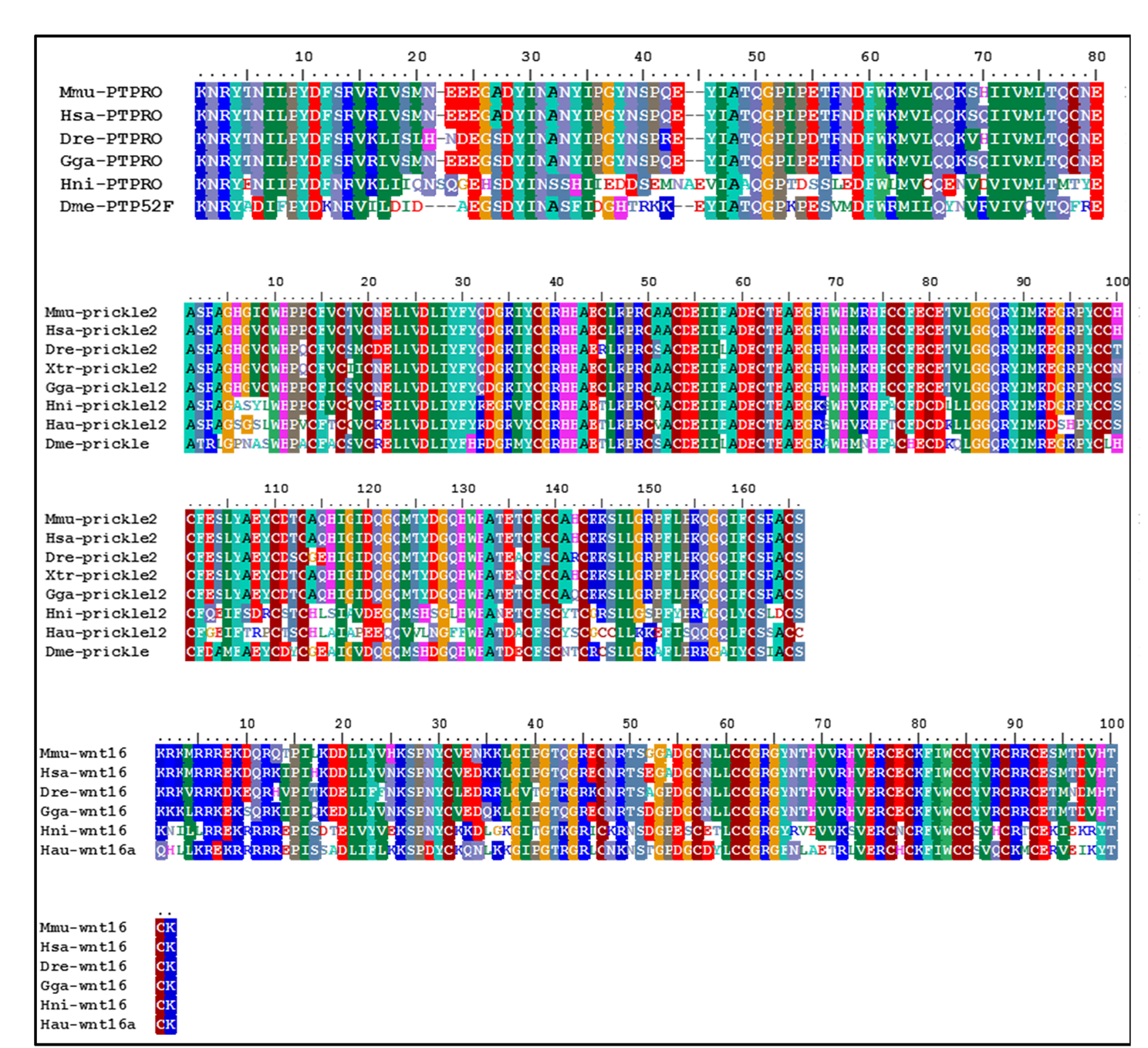
**

**Figure S1.** Protein alignment of PTPRO, PRICKLE2 and WNT16 showing conserved regions in diverse organisms including humans. Mmu, *Mus musculus*; Hsa, *Homo sapiens*; Dre, *Danio rerio*; Gga, *Gallus gallus*, Hni, *Hirudo nipponia*; Hau, *Helobdella austinensis*; Dme, *Drosophila melanogaster*.

**Table S1. Lists of primers used in the study**

| **Gene** | **Accession No.** | **Primer sequence** | | **Product Size**  **(bp)** |
| --- | --- | --- | --- | --- |
| *Prickle2* | MZ099648 | Forward | TGCTGCGTCTGCAGGGAGAT | 632 |
|  |  | Reverse | ACGTCTTGCTGCTGCTGCTG |  |
| *Ptpro* | MZ099650 | Forward | CATTTTCTTGGCAGACCATCAA | 323 |
|  |  | Reverse | GAAGGACCTAGAGAGTTATTTG |  |
| *Wnt16* | MZ099649 | Forward | GTCATTTACATGTGTATCTCTTTTC | 916 |
|  |  | Reverse | GCGAAGCGATTGGAAGTCG |  |

**Dataset 1.** Lists of genes identified with expression level (transcript per million, TPM) in mice teeth, *Hirudo* teeth and *Helobdella* proboscis.
